# Remarks on the Nature of the Changes Which Occur after the Extraction of the Natural Teeth, and the Propriety of the Early Introduction of Artificial Substitutes

**Published:** 1861-02

**Authors:** J. Richardson


### REMARKS ON THE NATURE OF THE CHANGES WHICH OCCUR AFTER THE EXTRACTION OF THE NATURAL TEETH, AND THE PROPRIETY OF THE EARLY INTRODUCTION OF ARTIFICIAL SUBSTITUTES.

(A Paper read before the Cincinnati Dental Association, November, 1860.)

BY PROF. J. RICHARDSON.

In a brief period after the extraction of the teeth, inflammation, of a higher or lower grade, is established, accompanied with some degree of tumefaction and tenderness in the parts implicated. If the inflammation is moderate in degree, and is permitted to run its usual course, it will be entirely consistent, in its ultimate consequences, with the requirements of the economy. If immoderate in degree and long continued, the natural processes or actions in the parts, determining the various changes which take place, will be more or less diverted or embarrassed.

The immediate issue or product of the inflammation which occurs is, fibrinous exudation from the ruptured surfaces of the periosteum of the socket, which, in its incipient stage of organization, takes the form of granules. In from ten days to two weeks, the socket is partially or entirely occupied with this fleshy substance. During this period, the actions are directed almost wholly to *reparation.* Absorption does not, ordinarily, occur until the inflammation has, in a great measure, subsided, and hence, at first, there is enlargement or tumefaction of the gums, rather than waste by absorption. As the inflammation subsides, however, these parts, which have no longer any special functions to perform, as the more protuberant portions of the alveoli and adherent gum, begin gradually to waste away under the action of the absorbents. Concurrently with this absorption, there is deposition of osseous material within the socket through some agency of the granules filling it, so that, in course of time, the cavity is partially filled in with bone analogous in structure with the maxilla proper, of which it ultimately forms a part; while the more superficial portions of the granular formations are transformed into tissues identical with the soft structures immediately surrounding. It is in this manner, by the concurrent and coöperating actions of absorption and deposition, that the maxillary ridges attain ultimately the smooth, regular and symmetrical form noticeable when the changes are completed.

In view of the facts, just advanced, the inquiry suggests itself, how soon after the extraction of the natural teeth may an artificial appliance be introduced into the mouth consistently with the conditions at first existing, and the integrity of the new formations?

We are amongst those who believe that the best interests of the patient require the early introduction of artficial substitutes, in all cases demanding full dentures. By so doing, we are enabled to confer immediate and signal benefits, by preserving, in a great measure, the customary expression of the individual,—promoting easy and distinct enunciation,—assisting in the more perfect comminution of food, and by maintaining unchanged the habitual relation of the jaws. The very general recognition of these important advantages has led to great unanimity of opinion in regard to the propriety and necessity of inserting what are termed *temporary* sets of artificial teeth.

More recently, the policy of inserting artificial teeth *immediately* after the extraction of the natural organs, is being advocated. Although the practice is seemingly heroic and hazardous, the reasons for its adoption are plausible. It is claimed that the inflamed parts, being shielded by a perfectly adapted plate, are less subject to injury in munching food, than when uncovered, the pressure in the former case being distributed over a larger surface, thereby equalizing the forces applied in mastication. Some good is also doubtless accomplished in these cases, by protecting the wounded and inflamed structures from the irritating action of the atmosphere and from sudden and direct impressions of extreme heat and cold.

On the other hand, it is held that the pressure of the base upon, and contact with, abraded surfaces already inflamed and sensitive, can not fail to aggravate existing morbid conditions. If such be the case, the practice may be fairly challenged; for any mode of procedure that would tend to increase the inflammation unavoidably present after the extraction of the teeth, or that would serve to extend it beyond the period of time when it should spontaneously subside, or that would interrupt, in any considerable degree, the reparative processes going on within the socket, is clearly inadmissible. Observation has fully confirmed us in the belief, that active inflammation in the structures about the sockets, retards absorption and delays the completion of those changes which it is important should occur at the earliest period consistent with the natural operations of the economy. We have frequently noticed, as we have no doubt all have, who have given the subject attention, that in cases where even moderate inflammation of the parts, either from local or constitutional causes, has continued long after the usual period for its subsidence, that little or no absorption has taken place. As well as we can remember, all of those cases of unusual tardiness, where the gums have remained almost wholly unchanged for a period of several weeks together, inflammation of a somewhat intractable character was found present, the gums remaining somewhat turgid, with diffused redness and some tenderness on pressure. Whether the causes operative are systemic or local, the condition manifestly opposes the action of the absorbents, their functions, for the time being, resting apparently in abeyance.

Our experience does not justify us in expressing a positive opinion as to the propriety or impropriety of inserting a plate at the earliest possible period after the removal of the natural organs. If solicited so to do, we should have no hesitation, after having extracted the teeth, to take the impression during the same sitting, and proceed with the operation at once, although it has been our usual practice to defer it until the more active stages of the inflammation, and the soreness consequent thereon, had, in a great measure, subsided.

In all cases, where the substitute is applied at an early period, the cavities in the model, corresponding with the sockets of the teeth, should be filled up even with the surface of the ridge, that the plate when swaged may not project into the sockets, and prevent, by mechanical obstruction, the normal development of the granular formations which are essential to the integrity of all the parts, and which can not be interrupted in any considerable degree without influencing the ultimate form of the jaw. It is in this manner that irregularities on the surface of the ridge are often produced, and not, as is erroneously supposed, by mere *pressure* of the base, the inequalities on the surface of which produce corresponding impressions upon the ridge.

